# Seroprevalence and Risk Factors Associated with *Chlamydia abortus* Infection in Sheep and Goats in North-Western Italy

**DOI:** 10.3390/ani14020291

**Published:** 2024-01-17

**Authors:** Chiara Nogarol, Monica Marchino, Sonia Scala, Manuela Belvedere, Giovanna Renna, Nicoletta Vitale, Maria Lucia Mandola

**Affiliations:** 1 Istituto Zooprofilattico Sperimentale del Piemonte, Liguria e Valle d’Aosta, S.S. Diagnostica Virologica Specialistica, Via Bologna 148, 10154 Torino, Italy; chiara.nogarol@izsto.it (C.N.);; 2 Servizio Veterinario ASL TO5, S.C. Sanità Animale, 10023 Chieri, Italy; marchino.monica@aslto5.piemonte.it; 3 Istituto Zooprofilattico Sperimentale del Piemonte, Liguria e Valle d’Aosta, S.S. Osservatorio delle Regioni, Via Bologna 148, 10154 Torino, Italy; nicoletta.vitale@izsto.it

**Keywords:** *Chlamydia abortus*, seroprevalence, risk factors, small ruminant, OEA

## Abstract

**Simple Summary:**

*Chlamydia abortus* is a pathogenic bacterium, belonging to the *Chlamydiales* family, able to induce abortion; sheep and goats, and less frequently cattle, pigs, and horses can be infected, but also pregnant women, representing a particular concern for human health. Its distribution may vary among regions and flocks worldwide due to well-defined risk factors. The aim of the present study is to fill the gap concerning the seroprevalence data of *Chlamydia abortus* in the Piedmont region (North-West of Italy), and to highlight the potential risk factors associated with its presence in small ruminants. During the study, the sera from 3045 sheep and goats belonging to 202 herds were tested. The study highlighted a high seroprevalence of *Chlamydia abortus* in small ruminant farms in the Piedmont region.

**Abstract:**

*Chlamydia abortus*, although poorly recognized as a human pathogen, is a zoonotic microorganism that can cause many different symptoms in humans, including subclinical infection and fatal illnesses in pregnant women. *C. abortus* is one of the most common causes of ovine and caprine infectious abortion worldwide, known as the causative agent of the enzootic abortion of ewes (EAE) or ovine enzootic abortion (OEA). To estimate *C. abortus* seroprevalence and the risk factors related to *C. abortus* in small ruminants, the sera from 3045 animals (both sheep and goat) belonging to 202 herds were tested and a questionnaire investigating flock management was administered. At the herd level, the true seroprevalence was 56.6% (CI_95%_: 46.9–66.3%), at sheep-farm and goat-farm level, the true seroprevalence was 71.4% (CI_95%_: 54.6–88.3%) and 44.8% (CI_95%_: 41.3–57.0%), respectively. The true seroprevalence was significantly higher among the sheep than the goats. The logistic regression model identified four factors associated with *Chlamydia* seropositivity: flock size (i.e., farms with >50 heads), contact with cattle, introduction of animals, and *Coxiella* seropositivity. The study evidenced a high seroprevalence of *Chlamydia abortus* in small ruminant farms in the Piedmont region. Considering its zoonotic potential and the health consequences in humans, communication to farmers on the importance of vaccination, as well as the sensibilization of farm vets, seem to be strategical.

## 1. Introduction

*Chlamydia abortus* (*C. abortus*) is a non-motile obligate intracellular Gram-negative pathogenic bacterium, belonging to the *Chlamydiales* family. Sheep and goats, and less frequently cattle, pigs, and horses can be infected by *C. abortus*; pregnant women can become infected by the microorganism, thus representing a particular concern for human health [1]. *C. abortus* is known as the causative agent of the enzootic abortion of ewes (EAE) or ovine enzootic abortion (OEA) [2], and along with other infectious agents, such as *Campylobacter* spp., *Salmonella* sp., *Listeria* sp., *C. burnetii* and *Toxoplasma* sp., represent the most common causes of ovine and caprine infectious abortion worldwide [3]. The distribution of these abortifacient pathogens may vary among regions and flocks worldwide due to the well-defined risk factors for these diseases, such as management practices, flock size, climatic conditions, vaccination, eradication strategies, or nutritional factors [4,5], although these characteristics do not represent the prevalence of the disease in different regions [6]. Furthermore, the relationship between agents and host to human transmission is influenced by many environmental factors [3]. *C. abortus* can survive in the environment from a few days to a few months thanks to the elementary body (EB) that can resist dry [7]. This resistance, as for *C. burnetii*, seems to increase the possibility of coming into contact with many animal species, farmed or wild, and with humans [1,3]. Referring to the literature data on PCR-based methods, co-infections in clinical cases of abortion are relatively frequent in small ruminants [8,9,10]. Taking the results published in an authors’ previous study on the seroprevalence of Q fever [11] structured on a well-defined study design to accurately estimate the prevalence of the infection [12] as a starting point and considering the direct evidence of co-infections based on biomolecular methods [8,9,10], in this study, we propose a retrospective seroprevalence study on sheep and goat serum samples.

Indeed, to the best of our knowledge, the recently published data are all related to *C. abortus* seroprevalence in small ruminants in non-EU countries [13,14,15,16,17,18]. In Europe, data about the caprine prevalence of OEA is scarce: some reports indicate a low seroprevalence in Slovak Republic (7.7%) [19] and in the Sardinia region, Italy (5.8%) [20], in contrast with a higher prevalence in Canary Islands, Spain (33%) [21]. The observed seroprevalence data are not so similar among studies, showing differences linked to animal breeds, farm management methods, sanitary conditions, and the serological test used. Indeed, the selection in most of the studies to employ an ELISA kit and an accurate estimate of the “true” prevalence adjusted for test sensitivity and specificity instead of the “apparent” prevalence is crucial in the light of harmonized and comparable data over time and between countries [11]. In this study, we use a commercial indirect ELISA kit with low cross-reaction between *C. abortus* and *C. pecorum* [22].

For this cross-sectional study, aimed to estimate the *C. abortus* seroprevalence in small ruminants in North-Western Italy, we selected non-vaccinated flocks along the provinces with higher farm density. Finally, the study also aims to determine the associated risk factors with *Chlamydia* seropositivity at the herd level.

## 2. Materials and Methods

### 2.1. Study Area

The Piedmont region (45°0′ N 08°0′ E, 25,399 km^2^) is located in the northwest of Italy, bordering Switzerland and France. The territory presents different landscapes with mountains, hills, and plains well-suited for the cultivation of cereal crops such as corn, wheat, and barley. A popular cultivation is rice, especially in the Vercelli province, where traditional rice fields cover a high percentage of the province territory. Piedmont livestock farming is mainly focused on cattle, goat, and sheep, both for meat and dairy production. Piedmontese cattle for meat and typical local cheeses like Toma and Robiola, are typical food products of this region.

Small ruminants farming may be considered representative of prealpine territories. The Italian National Livestock registration database (VetInfo, [23]) recorded, at animal level, 72,505 goats and 108,995 sheep. Among sheep, the Biellese is the typical breed, as well as the most relevant sheep bred in Italy. The prevalent breed among goats is the Alpine goat. At herd level, the farms registered in the region were 8288 (4573 goats, 1647 sheep, and 2078 mixed flocks).

### 2.2. Study Design

A cross-sectional study was conducted to estimate the seroprevalence of *Chlamydia abortus* at the farm and animal level in small herds of ruminants (sheep, goats and mixed) in northwestern Italy, between January and December 2012. The survey was planned with a two-stage design at farm level and animal level, setting a constant number of animals to be tested for each farm. Using the formula described by Cannon and Roe [24], the total number of sheep and goat farms to be sampled was selected considering an expected prevalence of 50%, with 7% precision at the 95% confidence level, with a design effect of 1.05. *C. abortus* vaccination was not applied to the selected target population. A proportional, stratified random sampling design was followed for the 202 flocks. Stratification was carried out by number of flocks per area. We select the number of animals to be sampled within flocks in order to detect at least one seropositive animal, considering an expected seroprevalence of 10% and a confidence level of 95% [25].

Blood samples were collected from small ruminants older than 6 months (i.e., minimum diagnostic age) by government veterinarians from the Local Health Units during annual mandatory brucellosis testing (related to the Brucellosis National Eradication Program). Information on flock management and potential risk factors for *Chlamydia* were registered using a questionnaire delivered to farmers by vets at the time of sampling.

### 2.3. Serological Test

Blood samples were collected from each animal and centrifuged (10 min, 1600× *g*). The obtained sera samples were stored at −20 °C ± 5 °C until analysis. A commercial indirect enzyme-linked immunosorbent assay (ELISA) kit was selected for the detection of IgG specific antibodies against *C. abortus* (ID Screen^®^ Chlamydophila abortus Indirect Multi-species, Innovative Diagnostic, Grabels, France). As declared in the kit instruction for use, the plates are coated with MOMP (Major Outer Membrane Protein) peptide, specific for anti-IgG *C. abortus* identification. The performances of the kit, as declared by the producer, are 70% (CI_95%_: 53.5–83.4%) for sensitivity and 100% for specificity (CI_95%_: 90.5–100%).

To obtain the optical density (OD) of each sample, the colored reaction was read in a microplate spectrophotometer at 450 nm. The percentage of reactivity of each sample was calculated using the suggested formula (OD_sample_/OD_positive control_) × 100, resulting in the sample classification as positive, doubtful, or negative. According to the cut-off established by the assay manufacturers, samples with a reactivity greater than 60% were classified as positive, those with reactivity between 50% and 60% as doubtful, and those with a reactivity lower than 50% as negative.

Serological data on *C. burnetii* seroprevalence were obtained as described in Rizzo et al., 2016 [11].

### 2.4. Statistical Analyses

Descriptive statistics were used to calculated the number of seropositive flocks for antibodies against *C. abortus*. As test specificity was 100% and no vaccination against *Chlamydia abortus* was used in the farms included in the study, a flock was classified as positive when at least one animal resulted positive to the test. According to Rogan and Gladen [26], the true seroprevalence was calculated using values of diagnostic accuracy given by the kit’s producer. A logistic regression model was used to estimate the association between serological response and potential risk factors for *C. abortus* to control for confounding. Statistical modelling was initially performed through bivariate analysis in order to select the relevant factors. The significance of factors in the model was tested using Wald’s χ^2^. As measures of the predictor effect, the estimated OR and 95% Wald’s confidence interval (CI_95%_) were calculated. Correlations between antibodies against *C. abortus* and the occurrence of other infections in the flock as *C. burnetii* were calculated using the Phi index. All of the analyses were conducted using SAS^®^ version 9.4 [27].

## 3. Results

**Figure 1:**
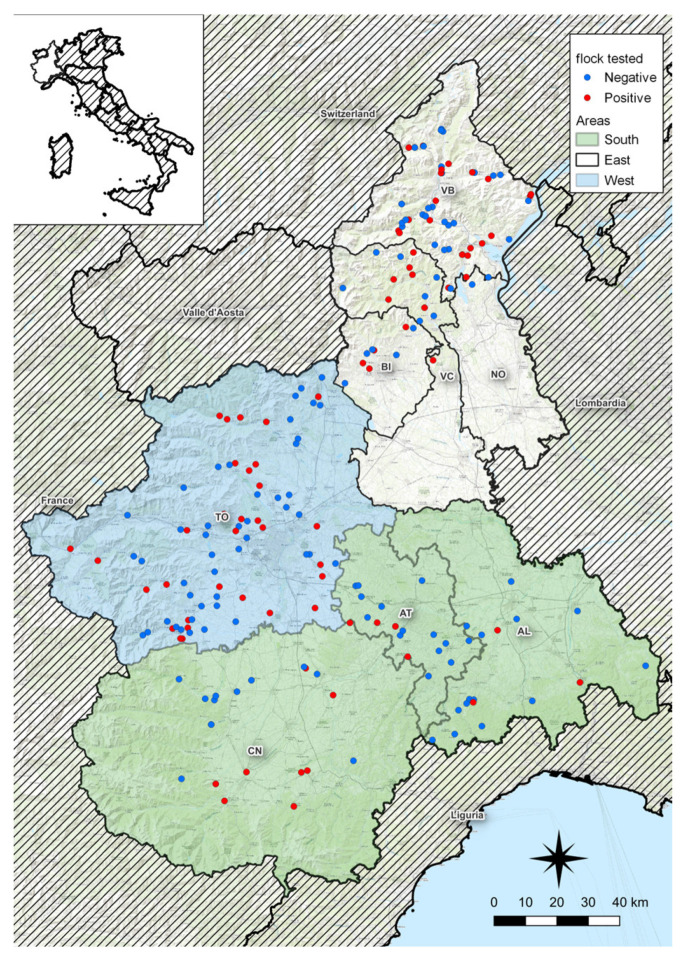
Spatial distribution of tested flocks according to *C. abortus* seropositivity and provinces. Red dots are positive flocks and blue dots are negative ones. No samplings were performed in the southern parts of VC and NO provinces because these areas are mainly devoted to rice cultures.

Sera from 3045 animals (1202 sheep and 1843 goats) belonging to 202 herds (118 goat herds, 72 sheep flocks, and 12 mixed herds) were examined for *C. abortus*. At the herd level, the true seroprevalence was 56.6% (CI_95%_: 46.9–66.3%); in detail, in the sheep-farm and goat-farm, the true seroprevalence was 71.4% (CI_95%_: 54.6–88.3%) and 44.8% (CI_95%_: 41.3–57.0%), respectively. Mixed herds, made up of both sheep and goats, show the highest true seroprevalence value (83.3%, CI_95%_: 36.6–100). On the other hand, considering the value of true seroprevalence at the animal level, the overall value is 22.3% (CI_95%_: 16.9–27.7%). In particular, these data were significantly higher (χ^2^ = 92.5; *p* < 0.0001) among sheep (33.4%, CI_95%_: 24.9–42%) than goats (15.1%, CI_95%_: 9–21.1%). The spatial distribution of the flocks according to *C. abortus* seroprevalence is shown in Figure 1. The true and raw seroprevalence at the farm and animal levels are shown in Table 1.

At the animal level, the factors associated with *C. abortus* seropositivity were species (χ^2^ = 91.5; *p* < 0.0001), age (χ^2^ = 10.2; *p* < 0.02), sex (χ^2^ = 7.2; *p* < 0.007), breed (χ^2^ = 43.9; *p* < 0.0001), and seropositivity to C. burnetii (χ^2^ = 27.9; *p* < 0.0001), as sheep, female, *C. burnetii* seropositive, mixed breed, and younger animals were more likely to be seropositive. Table 2 shows the results obtained using multivariate analysis (Wald chi square 103.6; *p* value < 0.0001). In particular, first kidding animals (1–2 years) (OR 1.6; CI_95%_ 1.2–2.3) showed a nearly two-fold increase in the probability of being seropositive compared with older animals, >4 years (OR 1). However, there was no correlation (phi = 0.09) between *C. abortus* and *C. burnetii* seropositivity at the animal level.

The factors associated with *C. abortus* seropositivity at the herd level are shown in Table 3. The main factors were flock size, introduction of animals, and contacts with cattle, *C. burnetii* seropositive. A moderate positive correlation (phi = 0.35) resulted between *C. abortus* and *C. burnetii* seropositivity at the herd level.

The results of the final logistic regression model (Likelihood Ratio χ^2^ = 44.6, degrees of freedom = 5, *p* < 0.001) are shown in Table 4. All of the studied factors were retained as being significantly associated with *C. abortus* seropositivity: flock size (i.e., farms with >50 heads) increased the probability nearly nineteen fold (OR 18.9; CI_95%_: 6.1–58.4); if we compare no contact or contact with bovine, the latter increased the probability of being *C. abortus* positive (OR 2.3; CI_95%_: 1.1–4.9) two-fold. *C. burnetii* seropositive flocks were more likely to also be *Chlamydia* positive, as *Coxiella* increased the probability of being *C. abortus* seropositive two-fold (OR 2.7; CI_95%_: 1.3–5.5). Finally, the introduction of new animals in the flock also doubles the probability of *C. abortus* seropositivity (OR 2.4; CI_95%_: 1.1–5.3).

## 4. Discussion

It is well-known that *C. abortus* is a zoonotic microorganism that can cause many different symptoms in humans, including subclinical infection and acute flu-like disease [2]. The pathogen can cause serious infections and fetal illnesses in pregnant women due to chlamydial-induced abortions [1,28]. In small ruminants farming, abortion and stillbirth represent important issues because of the financial impact caused by lamb and lactation loss. Many pathogens play a role in small ruminants’ abortion and reproductive failure, including *Brucella*, *Campylobacter* spp., *Salmonella* spp., *Listeria* spp., *C. burnetii*, and *Toxoplasma* sp.: the available word-wide data demonstrate that *C. abortus* is one of the most commonly diagnosed infectious causes of lamb loss [28]. Recent findings from the UK show that *C. abortus* was the main infectious agent responsible for reproductive disorders and fetopathy in 2021–2022 and the second one in 2023 [29].

In the Piedmont region, the Brucellosis National Eradication Program, implemented since 1992 (DM 453/1992), has contributed to eradicating the disease, obtaining disease-free status in 2009 (EU Decision 2009/342/CE del 23/04/2009). As for the other above-mentioned pathogens (excluding *Brucella*), *C. abortus* has not been the object of a similar national eradication plan; therefore, animals are not periodically tested for those specific pathogens. Reproductive failure or abortion may be investigated by farm vets based on farmers’ requests and/or symptoms and anamnestic outcomes. Consequently, no official epidemiological prevalence data are available. The aim of this retrospective study is to fill this lack of data in order to estimate seroprevalence in small ruminant farms in the Piedmont region (North-Western Italy).

In this study, about 40% of the tested farms resulted positive and the true seroprevalence was found to be even higher considering the specificity and sensibility of the ELISA test used. These data showed a wide spread of *C. abortus* in small ruminant farms in the Piedmont region; nevertheless, it is difficult to compare these results with other national and/or EU territories. The relevant data are not systematically collected, and often the only available information come from surveys and sporadic case-reports [3]. A previous study conducted between 1999 and 2003 in Sardinia (Italy) showed a low seroprevalence, with a rate of lower than 6% [20]. In neighboring Switzerland, a 19% *C. abortus* seroprevalence in small ruminant flocks was found, with great geographical variability, reaching a 41% seroprevalence rate in the Graubünden canton [30]. German studies described low seroprevalence in Saxony, as opposed to the 94% seroprevalence in the Thuringia region [31,32]. A Spanish study conducted in the Canary Islands on 325 goats from 11 non-vaccinated herds showed a 33% seroprevalence [21], while serological examination in sheep flocks in Belgium revealed a lower seroprevalence rate of 0.68% [33]. In contrast, Krkalic and colleagues described a 84.2% seroprevalence rate in sheep in Bosnia and Erzegovina territories [34].

A previous study conducted by the authors on small ruminants’ sera in Piedmont [11] revealed a *C. burnetii* seroprevalence comparable to that estimated for *C. abortus*, suggesting a possible co-infection for these two pathogens, as confirmed by the moderate positive correlation (phi = 0.35) found between *Chlamydia* and *Coxiella* seropositivity. In particular, the analysis of the risk factors associated with *Chlamydia* seropositivity suggest that, at the flock level, Q fever seropositivity doubled the probability of being *C. abortus* seropositive farm. This is in line with a study conducted in Spain and Portugal on 1600 small ruminant abortions: *C. burnetii* and *C. abortus* are the most common abortifacient agents, with approximately 75% of abortion cases testing positive for both pathogens, followed by *Toxoplasma gondii*, *Campylobacter* sp., *Salmonella enterica*, Border Disease virus, and *Neospora caninum* [35]. In addition, another Portuguese study described a relevant presence of co-infection of *C. abortus* and *C. burnetii* in small flocks of ruminants [10]. This association seems to be the same in large ruminants: a study conducted in Bosnia and Herzegovina in cattle dairy farms found a high correlation (gamma = 0.73, herd level) between *C. abortus* and *C. burnetii* co-infections. The authors concluded that the observed overlap between other agents were more random, both at the individual and herd level [36].

In this study, the value of true seroprevalence at the animal level was 22.3%, and the risk factors analysis showed that age (younger), sex (female), and species (sheep) increased the probability of being seropositive: younger animals, especially first kidding ones, tested positive more frequently. Indeed, protective immunity, which also prevents the re-occurrence of abortions, requires time to develop. The contact between ewe and the infected placenta induces a massive antigenic stimulation, followed by the instauration of the stimulated immunity response [3,37,38]. This is why, after their first abortion, ewes became resistant to future reproductive failure caused by *C. abortus*, even if they may become carriers and persistent shedders of *Chlamydiae*, especially during the estrus [39]. In addition to abortion, vaccination also confers protective immunity to infection [40].

Also, at the herd level, the seropositivity to Q fever seemed to be associated with the presence of *Chlamydia*; the other associated factors were flock size, introduction of new animals, and contact with cattle. As for flock size, this factor should be considered as a proxy of herd management and a productive feature: small flocks, with less than 10 subjects, may usually be considered as being not profitable and poorly managed, with a reduced number of the introduction of animals and a low- or no-reproduction rate. In contrast, large flocks are the ones that produce newborns, lactations, milk, and cheese; in these farms, *Chlamydia* may circulate during reproductive seasons. It should be considered that, in Italy, diagnostic tests for *Chlamydia* infectious status certification are not mandatory and are not routinely applied by farm vets in cases of the introduction of new subjects in a flock. Therefore, the introduction of animals of unknown health status should be considered a great risk factor, even more for a pathogen like *C. abortus* with asymptomatic carriers and persistent infected hosts. To reduce the risk of *C. abortus* introduction in cases of animal replacement, at managing level, may be used closed flocks, negative and/or vaccinated ones [3].

Infected and aborting ewes excrete *Chlamydiae* in large amounts in the placenta, uterine discharges, and feces, and those biological products may contaminate pastures, in which elementary bodies may persist and play a role in infecting new subjects, including other ruminants and wild animals. The elementary bodies released into the environment have great resistance to unfavorable conditions: they may remain infectious for a few days during the spring season (mild temperatures), but can resist from weeks to months at lower temperatures during the winter season [3,28]. This prolonged persistence in the environment raises the risk of possible contact with animals and/or humans, especially vets and farm workers; mixed farming in mountain areas during spring and summer may be considered as a risk factor for *C. abortus* transmission. As suggested by previous studies [41,42], a vaccination program that supports strategies fighting enzootic abortions must be taken into consideration: to date, the efficacy of inactivated and/or attenuated vaccines has been questioned because they can reduce the incidence of abortions and *C. abortus* shedding at parturition, but they do not prevent it completely. Despite the different approaches in the design of new vaccines against *C. abortus* (vaccine based on recombinant antigens, DNA vaccine, subcellular vaccine), there is still a lack of safe and efficacious vaccines in terms of shedding and reproductive disorders prevention.

In the present study, no history of stillbirth, preterm deliveries, or reproductive disorders appeared to be associated with *Chlamydia* seropositivity in the tested farms. However, the percentage of positive herds that had a history of stillbirth (50%) and pre-term deliveries (60%) resulted in a higher percentage of positive herds than those without a history of stillbirth (38.8%) and pre-term deliveries (38.9%). It should be considered that public health authorities be notified of abortions; however, in practice, this does not happen routinely. For this reason, in the Piedmont region, the underestimation of abortions is predictable, especially in small ruminant extensive or semi-extensive managing farming systems.

The study evidenced a high seroprevalence of *C. abortus* in small ruminant farms in the Piedmont region. Considering its zoonotic potential and the health consequences in humans (mostly pregnant women), communication to healthcare personnel to perform thorough anamnesis in pregnant women in farming areas and to advise them to avoid contact with small ruminants seems to be strategical. of equal importance would be to raise awareness among farmers and farm vets of the importance of applying biosecurity measures on farms due to the direct and indirect forms of human transmission of the pathogen [43]. Moreover, future in-depth analysis in and outside the Piedmont region are necessary to evaluate the *C. abortus* spread in different territories. These data will be useful for implementing biosafety measures to reduce the impact of the pathogen on small ruminant production systems, as well as to reduce its zoonotic potential. Finally, promising new vaccine strategies may be based on chlamydial outer membrane complexes (COMCs), showing that the conformation of antigens on the chlamydial surface may be important in eliciting the required protective immune response.

## 5. Conclusions

This is the first investigation on the seroprevalence of *C. abortus* in sheep and goats in North-Western Italy. The study demonstrates a widespread, high level of antibodies to *C. abortus* in the small ruminant herds in the Piedmont Region associated with the presence of *C. burnetii*. These results could be used to raise awareness among farmers about the risk associated with *Chlamydia*, given its zoonotic potential, as well as to minimize the risk of *C. abortus* infection in flocks under similar management in other areas. The seroprevalence of *C. abortus* may be overestimated in terms of the antigenic cross-reactivity between *Chlamydia* species, which may also operate as co-infections in the same herd or in the same animal. Therefore, planned follow-up studies using molecular tests will presumably improve our knowledge regarding chlamydial infection in these species.

## Figures and Tables

**Table 1:** Estimates of *C. abortus* seroprevalence at the flock level and the animal level for each ruminant species. At the flock level, true seroprevalence was adjusted by flock sensitivity and flock specificity, using Rogan-Gladen CL.

	Frequency (n)	Positive (n)	Raw Seroprevalence (CI_95%_)	True Seroprevalence (CI_95%_)
Flock level	202	80	39.6% (32.8–46.4%)	56.6% (46.9–66.3%)
Goat	118	37	31.4% (28.9–39.9%)	44.8% (41.3–57.0%)
Sheep	72	36	50.0% (38.2–61.8%)	71.4% (54.6–88.3%)
Mixed	12	7	58.3% (25.6–91.1%)	83.3% (36.6–100%)
Animal level				
Goats	1860	196	10.5% (6.3–14.8%)	15.1% (9–21.1%)
Sheep	1202	281	23.4% (17.4–29.4%)	33.4% (24.9–42%)
Total animals	3062	477	15.6% (11.8–19.4%)	22.3% (16.9–27.7%)

**Table 2:** Association between animal characteristics and *C. abortus* serological status with corresponding chi square, *p*-value, odds ratio (OR), confidence interval (CI_95%_).

Factors		Estimate	Adjuste OR	Wald CI_95%_		chisq	*p* Value
SPECIE	sheep	0.7680	2.155	1.687	2.754	113.4917	<0.0001
age	0 ≤ 1	−0.0311	0.969	0.522	1.802	37.7271	<0.0001
age	1 ≤ 2	0.4959	1.642	1.197	2.251	0.0097	0.9217
age	>2–4	0.4100	1.507	1.126	2.016	9.4808	0.0021
sex	F	0.9378	2.554	1.353	4.823	7.6104	0.0058
Breed	Other	0.7322	2.080	1.599	2.706	8.3633	0.0038
*Coxiella*	Positive	0.5583	1.748	1.279	2.389	29.7540	<0.0001

**Table 3:** Univariate analysis of farm-based factors associated with *C. abortus* seropositivity from 202 sampled farms. Significant *p*-values are given in bold.

Factors	Category	N° Flocks	Seroprevalence	Chisq	*p* Value
Flock location	North	81	43.20%		
	South	58	31.00%	2.3434	0.3098
	West	77	41.60%		
Flock composition	Goat	85	30.60%		
	Goat and Sheep	92	46.70%	4.8847	0.087
	Sheep	39	41.00%		
Flock size	Large (>50)	46	78.30%		
	Medium (10–50)	95	40.00%	48.3453	<0.0001
	Small (<10)	75	14.70%		
Production	Meat	99	36.40%		
	Others	99	39.40%	2.3507	0.3087
	Milk	18	55.60%		
Cheese production	No	199	38.70%	0.4592	0.498
	Yes	17	47.10%		
Summer pasture	No	86	34.90%	1.1953	0.2743
	Yes	130	42.30%		
Grazing systems	Sedentary	211	38.40%	3.5436	0.0598
	Transhumant	5	80.00%		
Introduction previous year	No	163	34.40%	6.9476	0.0084
	Yes	53	54.70%		
Contacts with cattle	No	158	32.90%	10.2267	0.0014
	Yes	58	56.90%		
Presence of dog	No	46	28.30%	3.0125	0.0826
	Yes	170	42.40%		
Contact with poultry	No	209	39.71%	0.3523	0.5528
	Yes	7	28.57%		
Contact with wildbirds	No	150	39.33%	0.0001	0.9933
	Yes	66	39.39%		
Contact with others flocks	No	141	34.80%	3.6005	0.0578
	Yes	75	48.00%		
Contact with workers of others flocks	No	175	35.40%	5.946	0.0148
	Yes	41	56.10%		
History of stillbirth	No	206	38.80%	0.4981	0.4803
	Yes	10	50.00%		
Preterm deliveries	No	211	38.90%	0.9144	0.339
	Yes	5	60.00%		
Reproductive disorders	No	115	39.10%	0.0051	0.9433
	Yes	101	39.60%		
*Coxiella* status	Unknown	17	58.80%		
	Seronegative	113	23.00%	23.993	<0.0001
	Seropositive	86	57.00%		

**Table 4:** Final multivariate logistic regression model for the presence of *C. abortus* antibody in the sera of 202 goats and sheep farms.

Parameter	Baseline	OR	CI_95%_	Wald χ^2^	*p*-Value χ^2^
Flock size Large (>50 heads)	small (<10)	18.9	6.1–58.4	26	0.0001
Flock size Medium (10–50)		4	1.7–9.8	9.6	0.002
Coinfection with *C. burnetii*	Positive	2.7	1.3–5.5	7.5	0.006
Introduction the previous month	Yes	2.4	1.1–5.3	5	0.03
Contacts with cattle	Yes	2.3	1.1–4.9	4.7	0.03

## Data Availability

The data presented in this study are available on request from the corresponding author.
